# Surgical treatment of a case of recurrent irradiated basal cell carcinoma of the head with a large soft tissue and bone defect

**Published:** 2009

**Authors:** Florescu Ioan Petre, Carmen Giuglea, Turcu Eugen, Marinescu Silviu, Jecan Crenguta, Mircea Gorgan, Bucur Narcisa

**Affiliations:** * Plastic Surgery Department, Clinical Emergency Hospital Bagdasar-Arseni, Bucharest; ** Neurosurgery Department, Clinical Emergency Hospital Bagdasar-Arseni, Bucharest

## Abstract

Taking into account the great number of skin malignances that occur in the head and neck regions, problems related to their surgical treatment represent a constant concern for plastic surgeons. They have to deal with the difficulties of radical excision and also with reconstructive possibilities. More than 2/3 of these malignances are basal cell carcinomas, which, if left untreated might become very invasive, surgical treatment being more difficult in such cases. Recurrent carcinomas combined with radiation injuries represent a serious challenge even for experienced surgeons regarding the size of the defect and anatomical structures involved. In this paper we present a case of a patient with basal cell carcinoma of the parietal region and the reconstructive treatment involving neurosurgery and plastic surgery team approach, using microsurgery techniques. We also present the difficulties of the case as well as the final outcome that we consider the best option for the patient. The neurosurgical stage consisted in fongus removal, excluding all brain tissue located outside the dural limits, craniectomy, duraplasty and cranioplasty. Because of the large size of the defect and also because the local resources were exhausted we chose as a covering solution the free tissue transfer consisting in a latissimus dorsi musculocutaneous flap. The difficulties of these cases consist both in the aim of radical excision and the limited reconstructive options. In this case, our collaboration with the neurosurgery team proved to be crucial, permitting us to solve this case in a single operative time, with deep excision, reconstruction of the dura mater and cranioplasty, and reconstruction of the soft tissues with microsurgical free transfer.

## Introduction

Taking into account the great number of skin malignances that occur in the head and neck regions, problems related to their surgical treatment represent a constant concern for plastic surgeons. They have to deal with the difficulties of radical excision and also with reconstructive possibilities. More than 2/3 of these malignances are basal cell carcinomas, which, if left untreated might become very invasive, surgical treatment being more difficult in such cases. Recurrent carcinomas combined with radiation injuries represent a serious challenge even for experienced surgeons regarding the size of the defect and anatomical structures involved.

## Case presentation

We are presenting a case of a 68 year old patient, who presented in our clinic with a basal cell carcinoma of the parietal region in 2004. He suffered multiple operations consisting in excision and cover with local flaps and skin grafts. Nowadays, he presented with local recidiva, necrotic infection in the exposed calvaria and a fungoid mass in the frontal part of the brain.

**Fig. 1:**
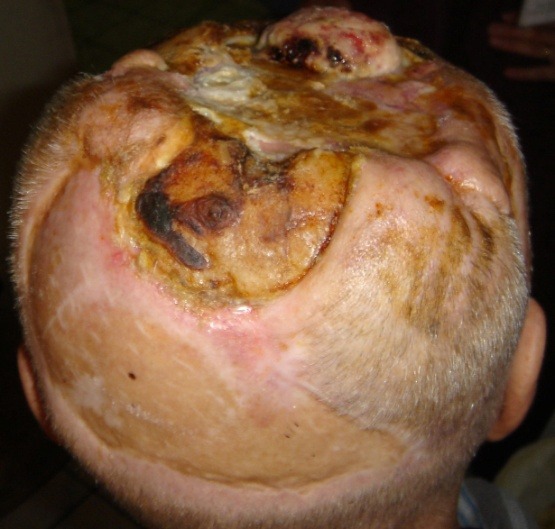
Preoperative aspects

**Fig. 2:**
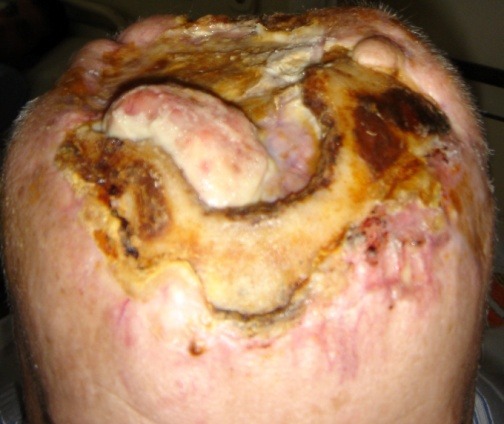


**Fig. 3:**
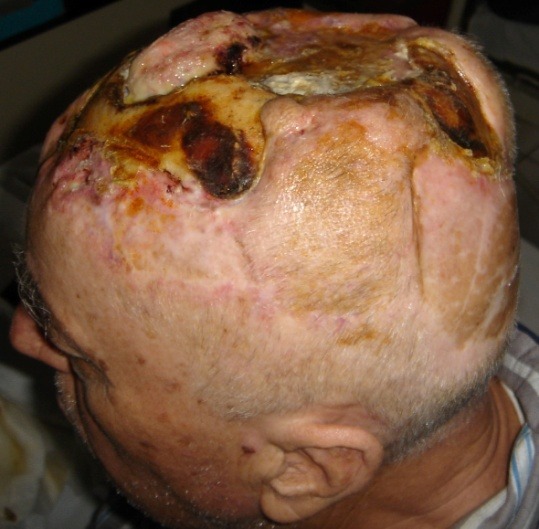


After clinical and paraclinical investigations, including CT and MRI, and also careful preoperative preparation, we performed the operation together with the neurosurgery team.

**Fig. 4:**
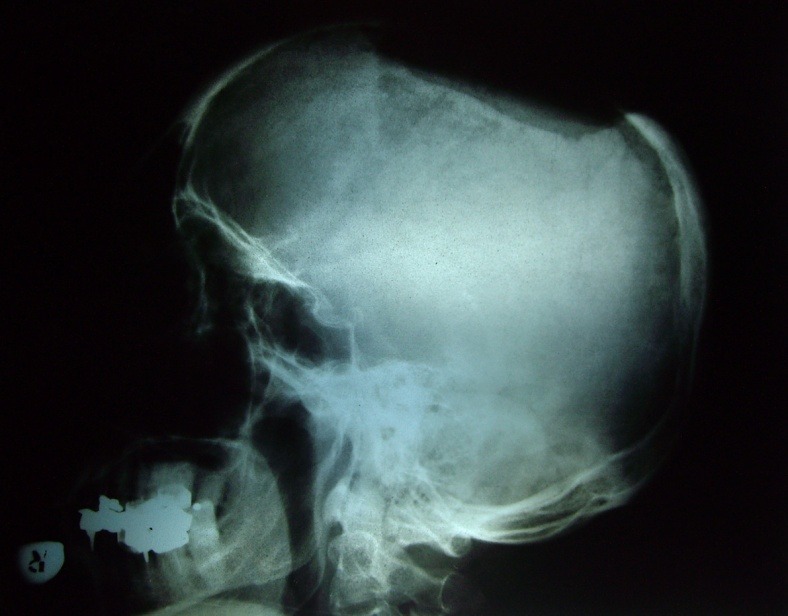


**Fig. 5:**
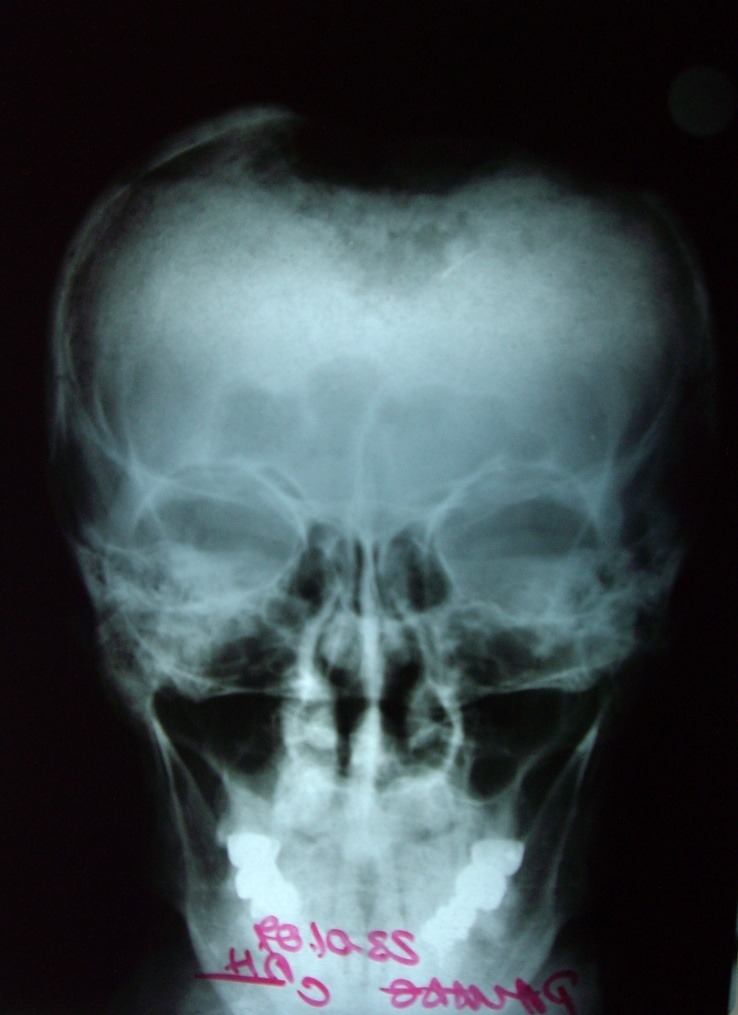


**Fig. 6:**
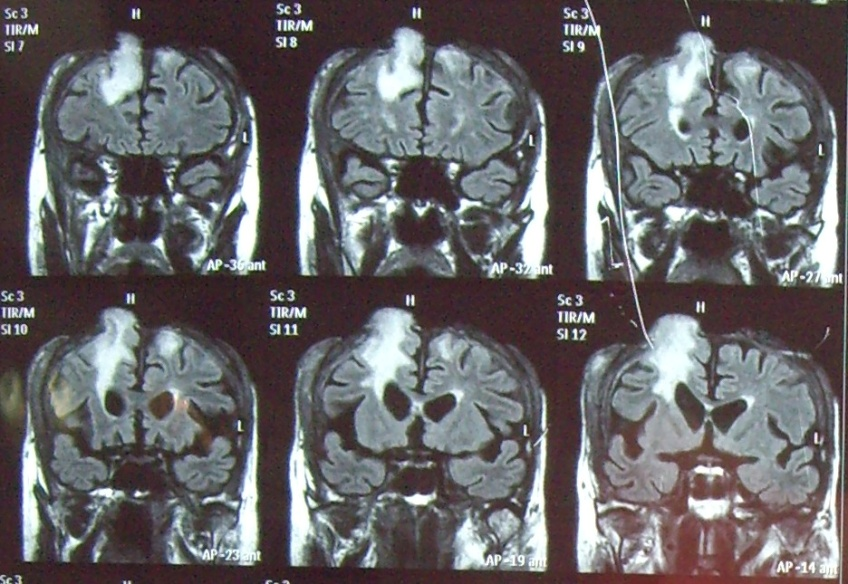


**Fig. 7:**
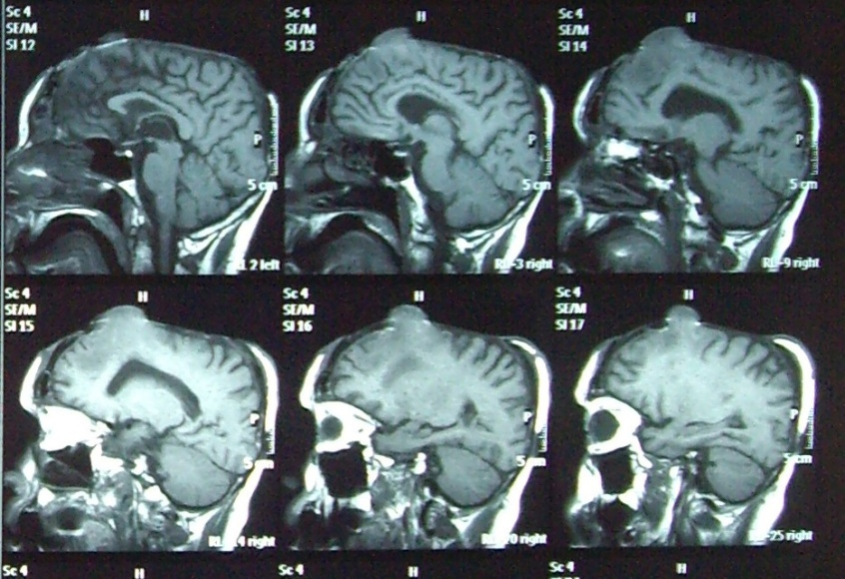


**Fig. 8:**
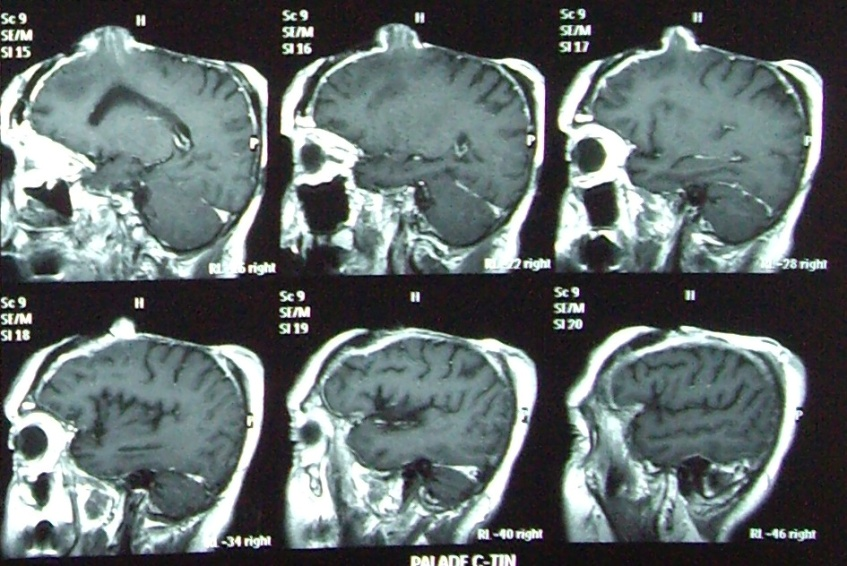


The neurosurgical stage of the operation began under general anesthesia with orotracheal intubation, and consisted in the next events:

A rigorous mechanical and chemical cleaning on the exposed surfaces, with peroxide and povidone iodine, in order to displace all debris and to appreciate exactly the limit of the living tissue. The inspection shows a tiny layer of dura mater, covering the superior sagital sinus and the convex aspects of both hemispheres, and a cerebral fongoid mass located on the right side, frontally, of about 3.5x5.5x3cm (see MRI images).

The bony defect of the calvaria was carefully inspected. The pericranium and the remnants of the scalp were detached circularly and isolated with cotton pads. The calvarial resection progressed from the frontal bone to the occipital, bilaterally, and with the aid of a Gouge rongeur the bone was sacrificed until bleeding, as the normal bone, was actually increasing significantly the defect to safe margins. The removed pieces were infected, necrotic and presented many micro-abscesses. The normal dura mater was exposed below the bony resection.

A new cleaning procedure with iodine was applied, especially on the bony edge.

The next step consisted of fongus removal, excluding all brain tissue located outside of the dural limits, proceeding to an adequate hemostasis, but not opening the subdural spaces (this method prevents CSF further leakage or fistula).

The entire surface of the defect was thereafter, covered with Duraform®, and abundantly placed over all. Saline 9 ‰ was used for humidification. Another layer of dural substitute made from cabalin pericardium, Neuropatch®, was prepared and applied over Duraform®, to seal any potential source of CSF leakage.

The last step was a cranioplasty, made from acrylic radio-opaque cement, Aminofix ®, adjusted on the place, which entirely covered the defect bone. The cranioplasty was fixed with the aid of Nylon 5 stitches, tractioned from the healthy parts of the pericranium, in an X shaped manner.

**Fig. 9:**
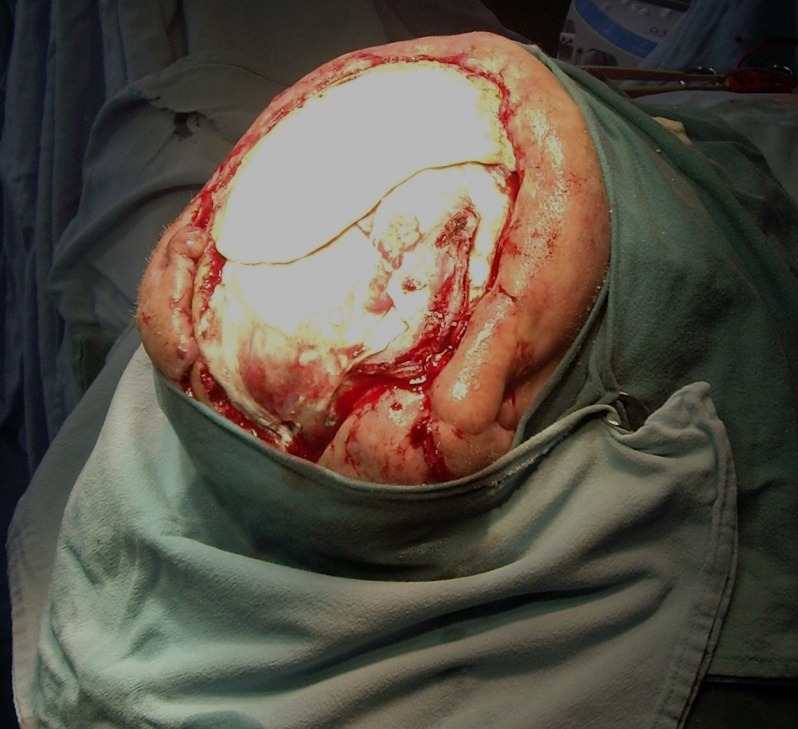


**Fig. 10:**
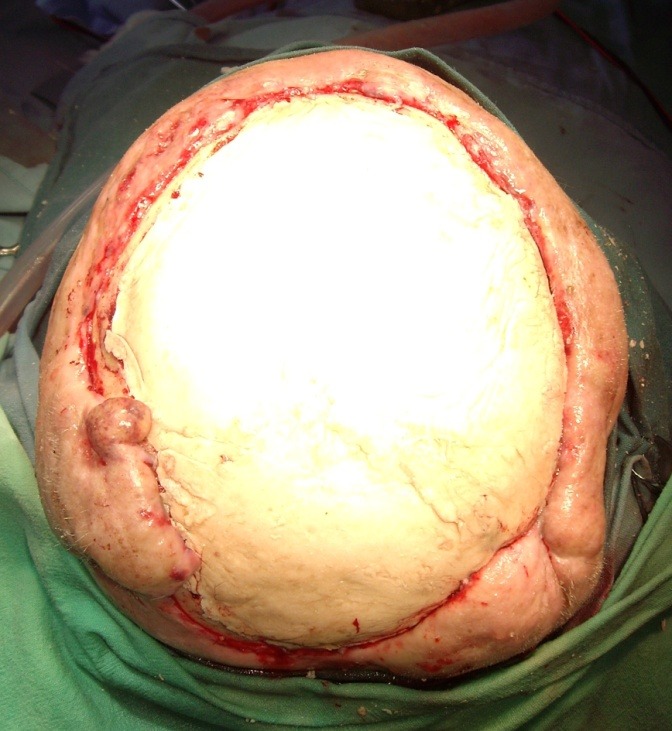


Because of the large size of the defect and also because the local resources were exhausted we chose the free tissue transfer consisting in a latissimus dorsi musculocutaneous flap as a covering solution. The criteria for choosing this flap were: the large size of the defect, the great reliability of this flap considering the radiation injuries in the recipient area and also less morbidity of the donor area.

The flap was harvested from the left side of the back, and was about 35 cm long and 25 cm wide, with careful dissection of the toracodorsal pedicle and also the pedicle for serratus anterior muscle. 

**Fig. 11:**
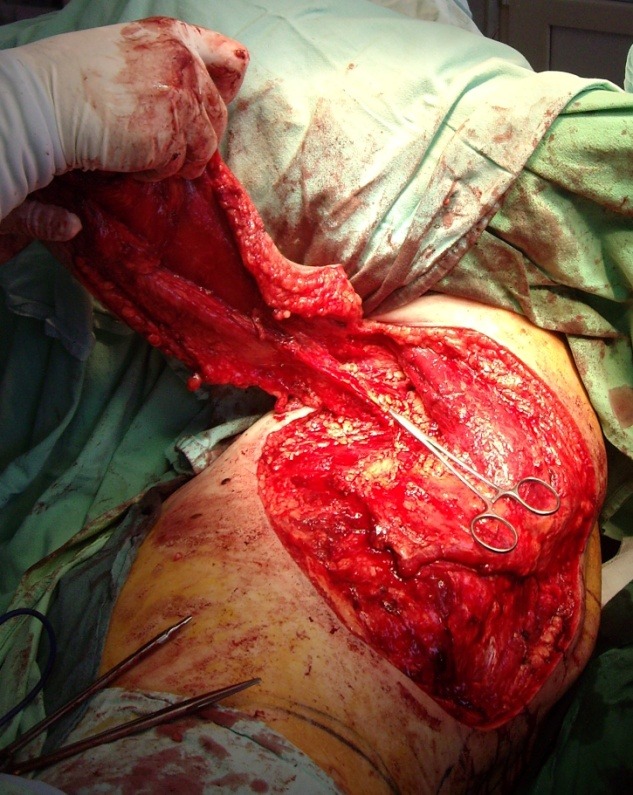


After harvesting, the flap was sutured in the new position and the vascular anastomoses were performed in the next manner: the toracodorsal artery was sutured end to end to the left superficial temporal artery, with atraumatic 8.0 wires, the toracodorsal vein was sutured end to end to a commitant vein of the superficial temporal artery and the vein from the pedicle for serratus anterior was sutured to another commitant vein. We decided to perform two venous anastomoses taking into account the large size of the flap, and also the bad condition of the recipient vessels.

The microsurgical stage of the operation took place without major problems, both arterial and venous anastomoses were patent and the flap was sutured to cover the defect. The donor area of the flap was closed with skin grafts.

**Fig. 12:**
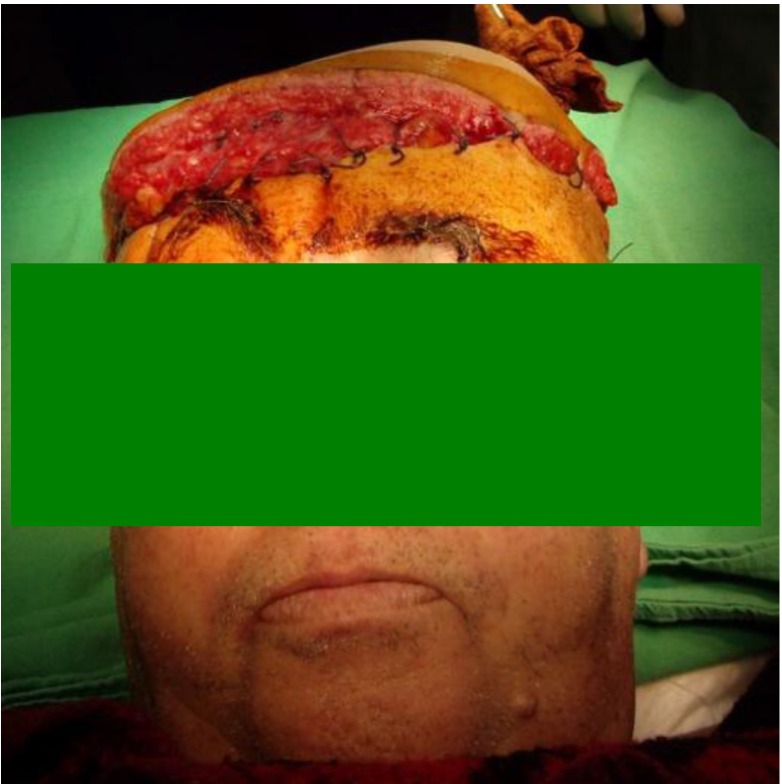


**Fig. 13:**
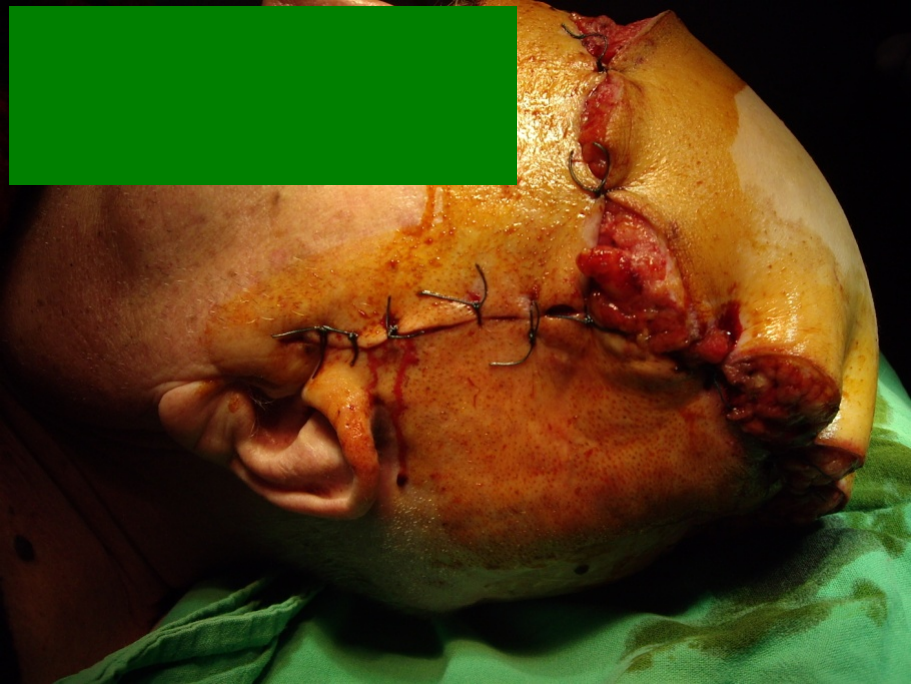


The local postoperative evolution was good, without major complications, only with a short period (a few hours) of venous congestion of the flap. In the next days the flap showed no signs of vascular suffering, with complete integration. 

**Fig. 14:**
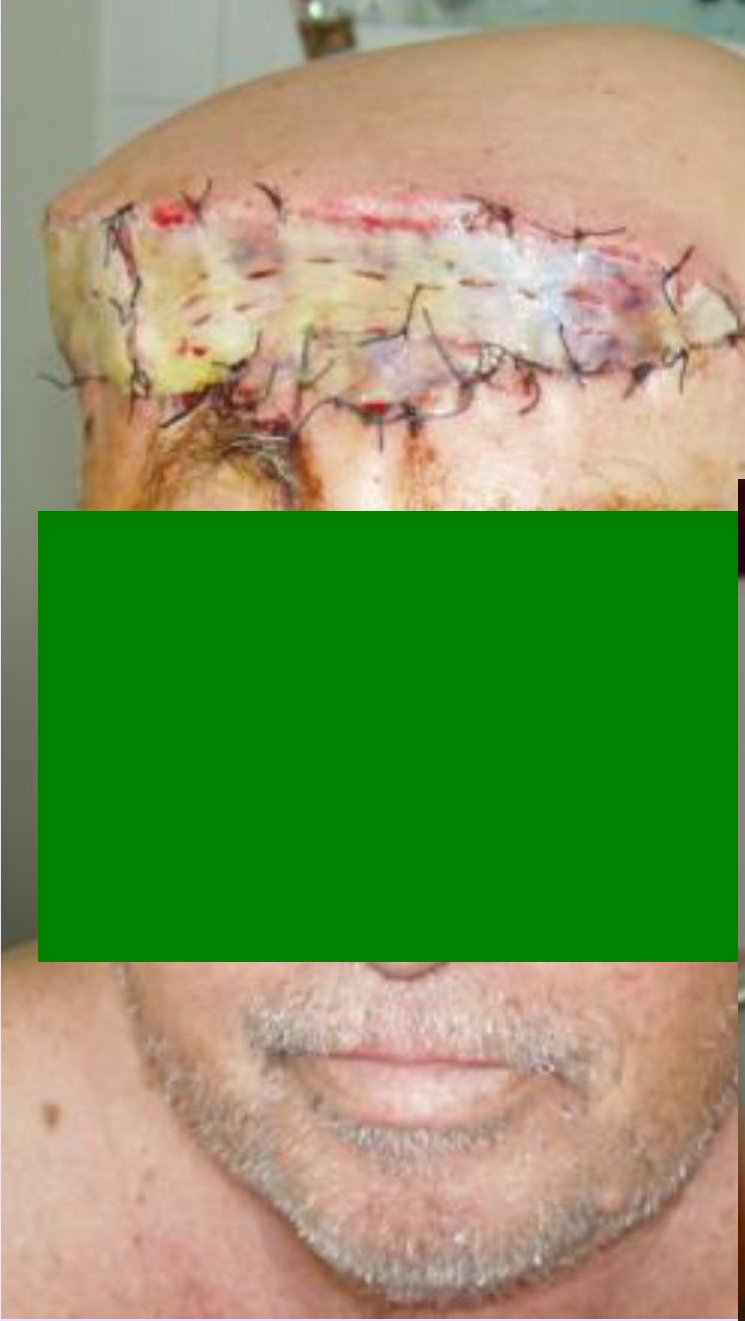


**Fig. 15:**
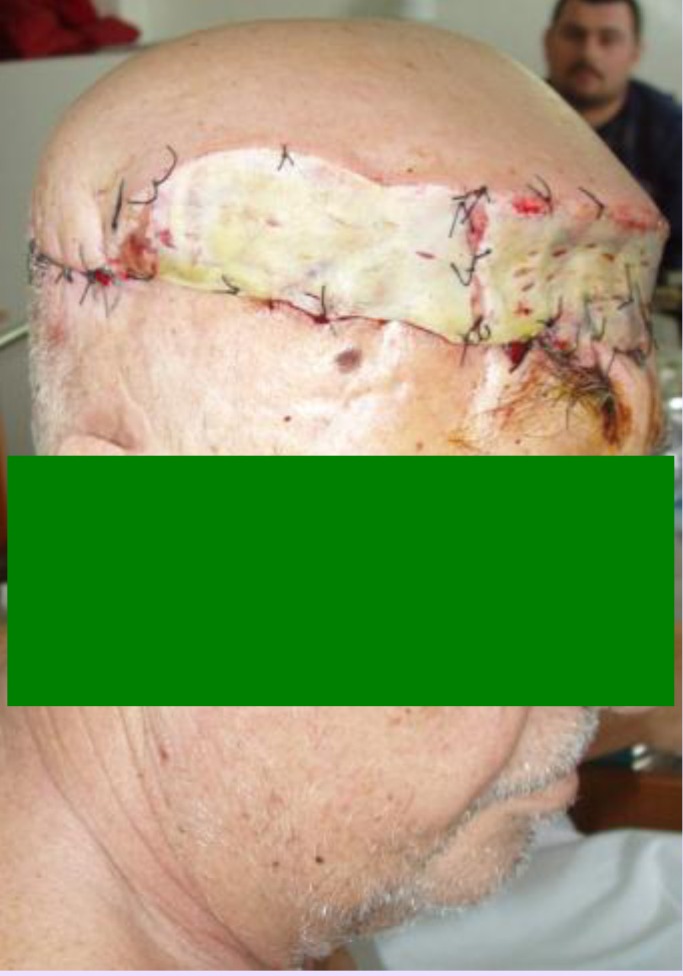


**Fig. 16:**
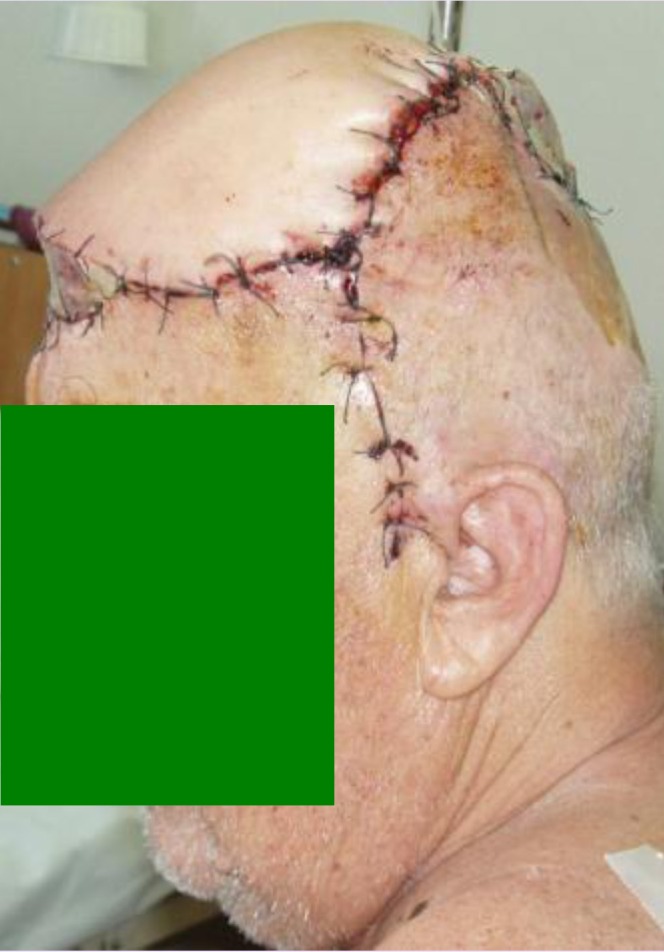


**Fig. 17:**
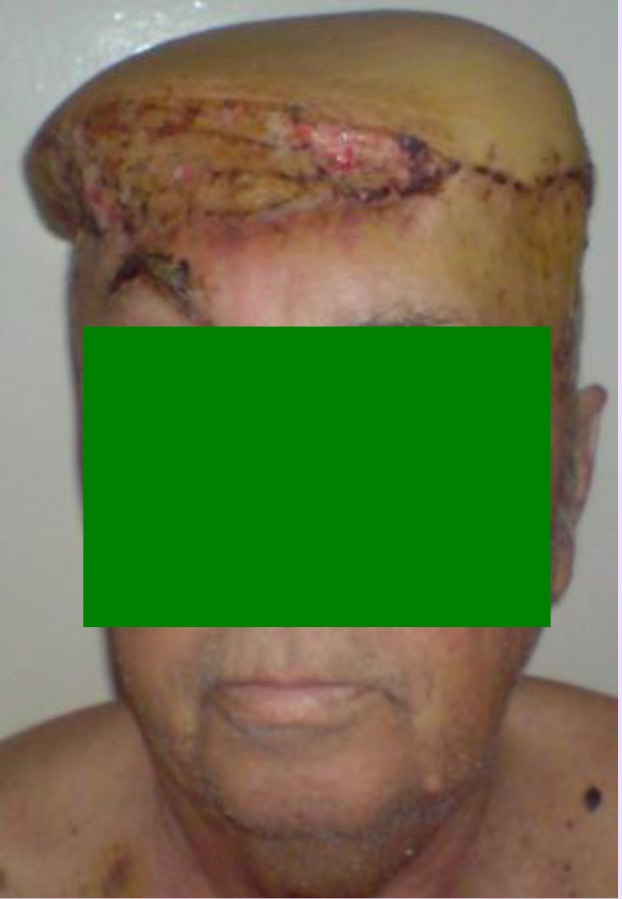


**Fig. 18:**
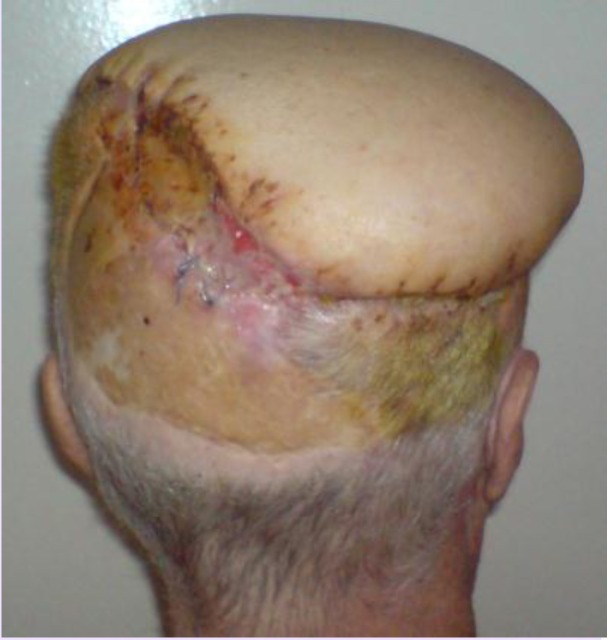


**Fig. 19:**
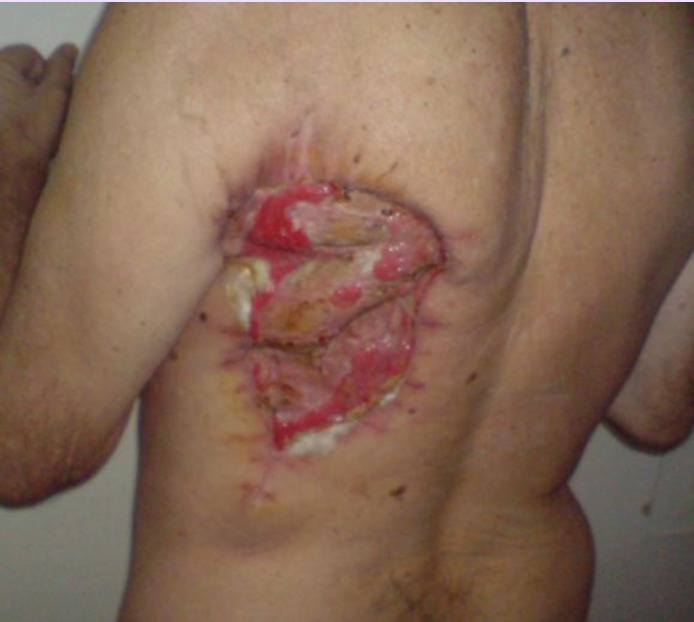


Postoperative check-up consisted of general monitoring of the patient, blood tests, ESR, etc. He received Avelox® 400 mg a day, for 21 days, in order to cover any septic potential complications to an irradiated and potentially immuno-compromised patient.

The meningism signs and thermal curve were controlled twice a day. A CT scan was ordered in the third day, and in the 14th day after operation. Images were evaluated, and compared, and blood or fluid collection inside the skull was excluded. Cranioplasty acrylic plate remains on place. A right frontal paraventricular hypodense area, below the operated fongus, remains as the previous, before surgery. Patient presents a moderate frontal syndrome, unchanged, as before surgery, without any motor or sensory deficit, in direct relation with surgery.

## Conclusions

Recurrent, irradiated basal cell carcinomas of the head which involve both skin and subjacent structures (including bone, dura mater and brain tissue) need large excisions, followed by large defects with serious covering problems.

Difficulties of these cases consist both in the aim of radical excision and the limited reconstructive options.

In this case, our collaboration with the neurosurgery team proved to be crucial, permitting us to solve this case in a single operative time, with deep excision, reconstruction of the dura mater and cranioplasty, and reconstruction of the soft tissues with microsurgical free transfer.
